# Pharmacological Applications and Action Mechanisms of Phytochemicals as Alternatives to Antibiotics in Pig Production

**DOI:** 10.3389/fimmu.2021.798553

**Published:** 2021-12-09

**Authors:** Lexing Li, Xueyan Sun, Dai Zhao, Hanchuan Dai

**Affiliations:** ^1^ College of Veterinary Medicine, Huazhong Agricultural University, Wuhan, China; ^2^ College of Food Science and Technology, Huazhong Agricultural University, Wuhan, China

**Keywords:** pharmacological applications and action mechanisms, alternatives, antibiotics, pig production, phytochemicals

## Abstract

Antibiotics are widely used for infectious diseases and feed additives for animal health and growth. Antibiotic resistant caused by overuse of antibiotics poses a global health threat. It is urgent to choose safe and environment-friendly alternatives to antibiotics to promote the ecological sustainable development of the pig industry. Phytochemicals are characterized by little residue, no resistance, and minimal side effects and have been reported to improve animal health and growth performance in pigs, which may become a promising additive in pig production. This paper summarizes the biological functions of recent studies of phytochemicals on growth performance, metabolism, antioxidative capacity, gut microbiota, intestinal mucosa barrier, antiviral, antimicrobial, immunomodulatory, detoxification of mycotoxins, as well as their action mechanisms in pig production. The review may provide the theoretical basis for the application of phytochemicals functioning as alternative antibiotic additives in the pig industry.

## Introduction

Antibiotics are natural products or derivatives of natural products, and widely used for infectious diseases and feed additives for animal health and growth over the past decades (1). Antibiotics may lose effectiveness against a growing number of bacterial pathogens, reducing competition and expanding the resources available to resistant bacteria, which results in antibiotic resistance (AR) (2, 3). AR is spreading rapidly because once a resistance gene evolves in one bacterium, it can spread to other cells and other bacterial species (3, 4). AR accounts for hundreds of thousands of deaths annually, and its wider implications present us with a growing global health threat (5). Development of AR and measures to combat AR have become important issues that are not counterbalanced by the development of new therapeutic agents (6, 7).

Pork is considered as a good and cheap alternative source of animal protein and a rich source of B complex vitamins (8). The development of hog production does not only meet the demand of pork and its products, but also contribute a lot to the farmers’ income increase, rural labor employment, transformation of grains, and stimulating the development of related industries. However, pig breeding is endangered by a variety of infectious diseases. Pork and its products not only infect people with bacterium disease, but also viral diseases, parasitic diseases and spiral diseases (9, 10). The unnecessary use of antibiotics and concomitant rapid growth of AR is a widely acknowledged threat to the development, and sustainability of pig production. It is reported that antibiotic consumption in food animal production was conservatively estimated at 93,309 tons in 2017. Globally, sales are expected to increase by 11.5% in 2030 to 104,079 tons (11). Overuse of antibiotics results in increasing levels of AR and food residues in the pig industry, and the arsenal of effective antibiotics is diminishing, which lead to severe challenges in food safety, animal health, and environmental protection (12, 13). World Health Organization (WHO) has declared it a risk for both human and animal health (14). Many countries have approved the withdrawal of colistin as a feed additive in animals, including Brazil (November, 2016), Thailand (February, 2017), China (April, 2017), Japan (July, 2018), Malaysia (January, 2019), Argentina (February, 2019), and India (July, 2019) (15, 16). Therefore, alternative measures need to be sought to maintain swine health and performance. Some alternatives including plant extracts, essential oils, probiotics, prebiotics, symbiotic, dietary fiber and enzymes, antimicrobial peptides, and functional amino acids have been used as feed alternatives to improve health and growth performance in pig industry (17–19).

Phytochemicals have been used for many years to treat various ailments. Some of the compounds of plant origin such as phenolic compounds (*apigenin, quercetin, curcumin and resveratrol*), organosulfur compounds (*allicin*), terpenes (*eugenol*, *thymol*, *carvacrol*, *capsaicin*, *and artemisinin*) and aldehydes (*cinnamaldehyde and vanillin*) have different properties such as antimicrobial (including antibacterial, antifungal, antiviral and antiprotozoal), antioxidant, immunomodulatory, and detoxification of mycotoxins, as well as the improvement of intestinal morphology and integrity of the intestinal mucosa (20–22). Phytochemicals are characterized by little residue, no resistance, and minimal side effects and serve as powerful therapeutics against pathogenic bacteria or act as functional additives, which have been reported to improve animal health and growth performance in pigs. Phytochemicals have great potential as substitutes for antibiotics, and may become a promising additive in pig production. The development of new antibiotics around proven natural scaffolds is the best short-term solution to the rising crisis of antibiotic resistance (23). In this article, we discuss the pharmacological applications of phytochemicals as alternatives to conventional antibiotics. The potential action mechanisms of phytochemicals in swine production are also reviewed. Some thoughts for future research and application are put forward to improve swine health.

## Pharmacological Applications of Phytochemicals in Pig Production

Phytochemicals are widely used in the animal production industry. The diet supplemented with phytochemicals can improve meat quality, increase the amino acid concentration of muscle, increase the villus height: crypt depth ratio, and induce positive effects on serum biochemical parameters and immune function in swine (24). For instance, *coix* seed (scientific name *Coix lacryma-jobi*), from a grain-bearing perennial tropical plant (25), has been used to fortify the spleen and inhibit dampness, significantly enhance the average weight gain, and reduce the feed/meat ratio in post-weaning pigs. *coix* seed administration decreases the pH value of gastric juice and elevates the density and length of gastrointestinal villi. *Coix* seed exerts a good potential growth performance effect in weaned pigs, which is associated with increased amounts of *Lactobacillus* and *Bacteroides* and the enrichment of microbial metabolic pathways. (26). Additionally, *wolfberry* is well known for its health benefits in Asian countries and has a positive effect on boar semen quality (27). Moreover, the active and safe compounds isolated from plants can additionally serve as drug leading for therapeutic purposes (28). Phytochemicals are volatile lipophilic compounds, which are constituted of a complex mixture of terpenoids and phenols, which function as antioxidant, antimicrobial, immunomodulatory effects, as well as improving intestinal morphology and integrity of the intestinal mucosa (29, 30).

### Effect of Phytochemicals on Pig Growth Performance

Due to the concerns regarding AR and antibiotic residues, antibiotic growth promoters have completely or partially banned to use in pig products in several countries. AR and residues are important factors related to animal growth and animal product quality. Natural bioactive compounds can prevent the animal growth check and enhance gastrointestinal health. Phytogenic feed additives have been widely used in view of the plant-derived properties and growth-promoting effect, which suggests that phytogenic feed additives will be a promising alternative to antibiotic growth promoters (21, 31). It is shown that the 300 ppm *Laminarin* group has a higher ADFI (average daily feed intake) and a higher ADG (average daily gain) than the basal group (32). *Laminarin* supplementation elevates villus height in the duodenum and jejunum, improves performance, and prevents post-weaning intestinal dysfunction. *Laminarin* exerts a positive influence on intestinal health through alterations in the gastrointestinal microbiome (32, 33). The results from research evaluating phytogenic additives as growth performance enhancers are variable (34). YGF251, extracted from herbs including *Phlomisumbrosa Turez*, *Cynancumwilfordii Hemsley*, *Zingiberofficinale Rosc*, and *Platycodi Radix*, positively promotes growth performance, nutrient digestibility, immune function, and fecal gas emission in pigs. However, Dietary supplementation with 0.05% herbal extract *YGF251* in low protein diets does not effectively improve growth performance (31). Studies show that supplementation of combined *Chestnut* (Ch) and *Quebracho* (Qu) increase the serum concentration of albumin and albumin/globulin ratio, but decreased creatinine, and did not influence growth performance (35). However, *tannins* extracted from Ch and Qu have been applied on intensive swine farms due to their ability to improve animal performance and health. These positive and prominent effects are frequently associated with the anti-inflammatory activity and antioxidant activities in weaning and post-weaned piglets (36, 37). Particularly, 500 and 1,000 mg/kg of microencapsulated *tannic acid* (TA) is safe to be included in the swine diet and that 1,000 mg/kg of microencapsulated TA has beneficial effects on intestinal morphology, intestinal nutrient transporter, and intestinal microbiota in weaning piglets. Microencapsulated TA functions as a suitable alternative to antibiotics for improving growth performance in weaning piglets (38). It is demonstrated that 0.3 g/kg *Eucommia ulmoides leaf* extracts (ELE) increase the average daily gain compared with basal diet and alkaline phosphatase (AKP) levels. Total antioxidant capacity (T-AOC) is elevated in serum and liver, accompanied with a higher villus height of the duodenum and jejunum (39). Besides, dietary supplementation of artificial sweetener and capsicum oleoresin can mitigate the negative consequences of heat stress on pig performance (40). Supplementation with *B. papyrifera* leaf extract can increase the growth performance and antioxidant capacity of weaned piglets (41). The effect of phytochemicals on growth performance elevation may associate with its antioxidant capacity or anti-inflammatory activity. Natural phytochemicals represent a promising non-antibiotic tool to allow better intestinal health, nutrient digestibility, and general health status, thereby leading to improve growth performance.

### Effect of Phytochemicals on Metabolism

Phytochemicals improve metabolism by regulating the expression of metabolism-related proteins and antioxidant stress-related proteins. Phytochemicals may exert beneficial health effects by regulating energy metabolism and activating enzymatic mechanisms, and may substitute dietary antibiotics. A paper reports that cinnamon bark extract contributes to improve glucose metabolism and lipid profile in fructose-fed rats (42). Also, oral supplementation with food-derived phospholipids has been associated with beneficial effects on lipid and lipoprotein metabolism (43).

It can be concluded that supplemented with 2% of inulin extract from chicory root or 4% of dried chicory root for 40 d significantly modulates the expression of liver proteins associated with energetic metabolism, particularly those involved in cholesterol and TG metabolism in growing pigs. Additionally, both dietary additives increase the expression of proteins related to hepatocyte protection against oxidative stress (44). Diet supplementation with dried *Chicory* root or inulin causes significant changes in the expression of liver cytoskeletal proteins, and alters the expression of kidney proteins engaged in energy metabolism and stress response in pigs (45). Moreover, *Chicory* root supplements have a potential ability to improve the liver mineral content oxidoreductive homeostasis in growing pigs (46). Particularly, a proteomic study shows that inulin-type fructans can enhance the anti-inflammatory properties by downregulation of hepatic acute phase protein CK18 in growing pigs (44). Sows fed with *Garcinol* (200 or 600 mg garcinol per kg) in late gestation and lactation (from the 90th day of pregnancy to day 21 postpartum) improve the maternal health and antioxidative status, milk protein content, acid–base balance in the umbilical cord blood, and growth performance in piglets (47). Reductions of the fat percentage in milk on day 7 and day 14 are found in sows supplemented with Oregano (OEO) treatment. Milk from sows supplemented with OEO during lactation has the greatest number of T lymphocytes, and a trend for greater milk intake is observed in piglets (48). Moreover, dietary supplementation with OEO can be effective in reducing the performance loss due to the outdoor-rearing system without modifying most of the peculiar traits of the meat, and improve growth rate and antioxidative status in outdoor-reared (49). *Black pepper* extract (BPE) supplementation has positively enhanced the growth performance, nutrient digestibility, fecal microbial, fecal gas emission, and meat quality of finishing pigs (50). *Betaine* 1250 mg/kg (Low Betaine) or 2500 mg/kg (High Betaine) may promote muscle fatty acid uptake *via* up-regulating genes related to fatty acid transporters including FAT/CD36, FATP1 and FABP3. On the other hand, *Betaine* activates AMPK and up-regulates genes related to fatty acid oxidation including PPARα and CPT1 in a lasting 42-days feeding experiment (51). 3.5% *linseed oil* (LSO) supplementation increases immunoglobulins, modifying the fatty acid composition. The concentration of 18: 3n-3 fatty acids is higher in the milk of LSO sows (gestation of day 107 to the lactation of 28th day) and n-3 polyunsaturated fatty acid (PUFA) in the tissues of piglets weaned on day 21 is increased. And the genes of D5D (Δ5-desaturase) and D6D (Δ6-desaturase) expression of piglets are affected (52). *Flaxseed* oil may have a positive effect on alleviating muscle protein loss and carbohydrate oxidation impairment induced by LPS challenge through regulation of TLR4/NOD and Akt/FOXO signaling pathways (53). Murtilla or murta (*Ugni molinae* Turcz), a plant of the Myrtaceae family, is rich in phenolic components. Murtilla extract (MT-ex) exerts antioxidant activity, and anti-inflammatory (54). However, MT-ex administration generates an important decrease in sperm metabolism, especially ATP production, which might affect the fertilization potential due to the reduction in sperm motility. Nevertheless, the metabolic decrease would allow greater efficiency in the refrigeration of boar semen at 17°C (8). Therefore, phytochemicals may play important roles in the metabolic regulation of proteins or genes associated with energetic metabolism, hepatocyte protection, fatty acid transporters, and oxidation. The metabolic effect of phytochemicals might be the key to explain the diverse pharmacological effects described in practical applications, which would contribute to a better understanding of its various functional properties in pig production.

### Effect of Phytochemicals on Antioxidative Capacity

Oxidant and oxidative stress accelerate the free radical generation resulting in cell, DNA, protein, and lipid damage and loss of biological function, which are linked to many diseases (55–57). Free radical mediated oxidative stress may play a decisive role in the pathogenesis and progression of chronic metabolic diseases. Anti-oxidative therapy has been proposed as a promising and effective strategy for preventing metabolic diseases (58, 59).

Antioxidant activity of phytochemicals is one of their most intensively investigated properties. Oxidation of many biological substances causes cellular damage and metabolic diseases (60). The decreased antioxidant capacity and reactive oxygen species (ROS) level elevation are involved in weaning-induced intestinal dysfunction, resulting in intestinal oxidative stress (61), which is frequently associated with inflammation, elevation of interleukin 1β (IL-1β), tumor necrosis factor α (TNF-α), and interleukin 6 (IL-6), decreasing the abundance of tight junction proteins, and undermining the integrity of the intestinal barrier. Excessive ROS exerts adverse effects on cells by reacting with phospholipids and oxidizing sulfhydryl groups in enzymes, proteins, and DNA.

It has been reported that OEO exerts antimicrobial and antioxidative ability to improve intestinal barrier function and growth in pigs. OEO supplementation to maternal rations during late gestation and lactation can lead to improvements in progeny health and performance with a reduced incidence of mortality and lower need for medication (62). The main active compounds carvacrol and thymol in OEO possess ROS-scavenging activity *in vitro*. Dietary supplementation with 100 mg/kg carvacrol–thymol (1:1) decreases the intestinal oxidative stress and influences selected microbial populations without changing the biomarkers of the intestinal barrier in weaning piglets (63). Another study reports that *thymol* (510 mg/kg feed) contributes to regulating the integrity of the intestinal mucosa because of their anti-inflammatory and antioxidant properties (64). Plant extract resveratrol attenuates oxidative stress-induced intestinal barrier injury through PI3K/Akt-mediated Nrf2 signaling pathway (65). However, due to the labile nature, volatiles, and being rapidly absorbed in the upper gastrointestinal tract, the stability of EO during feed processing is often questionable to result in a challenge. Here, a report demonstrates that lipid matrix microparticles are able to maintain the stability of thymol and allow a slow and progressive intestinal release of thymol in weaned pigs (66).

In terms of reproductive performance, *cWGRE* (cultured wild ginseng root extract) has positive effects on male reproductive function *via* suppression of ROS production (67). Supplementation with *astragalus polysaccharides* (APS), as well as *murtilla* extract (MT-ex), can effectively preserve sperm motility, acrosome integrity and mitochondrial membrane potential through inhibiting the protein dephosphorylation caused by ROS *via* cAMP-PKA signaling pathway. The compound can eliminate the excessive mitochondrial ROS, improve antioxidant capacities and enhance ATP levels, which is an advantage in extending the useful life of boar sperm (8). Besides, OEO dietary supplementation increases the retention of α-tocopherol and antioxidant capacity in spermatozoa of boars fed with a fish oil-fortified diet. Moreover, a feeding diet containing 500 mg/kg OEO greatly attenuates sperm membrane and DNA oxidative damage by reducing ROS production (68). *Grape seed procyanidin* B2 (GSPB2) inhibits oxidative stress-induced apoptosis of porcine ovarian granulosa cells through the increased let-7a (69). In conclusion, phytochemicals may activate a series of signal transduction to clear ROS, reduce apoptosis levels, suppress the expression level of inflammatory factors, maintain intestinal mucosal integrity and change oxidative stress levels, to maintain normal cell and body functions.

### Effect of Phytochemicals on Intestinal Mucosa Barrier

The gut is hypothesized to be the “motor” of critical illness and the intestinal epithelium absorbs nutrients, which may be as the first-line protection against pathogenic microbes and as the central coordinator of mucosal immunity to play roles (70, 71). The gut plays a dynamic role in organ integrity, immunity, and body defense against harmful antigens, toxins, and pathogens.

Weaning is a major critical period in pig husbandry, which involves complex dietary, social, and environmental stresses that interfere with gut development. Significant efforts are being made to identify natural alternatives to support homeostasis in the piglet gut, in particular during the weaning period (72).

Supplement with *Cynara scolymus extract* (CSE) for 15 days can be considered as a nutritional strategy to prevent enteric disorders and improve intestinal health in post-weaned piglets (73). 100 mg/kg *Forsythia suspensa* extract (FSE) or 160 mg/kg *chito-oligosaccharide* (COS) can increase performance by modulating intestinal permeability, antioxidant status and immune function in younger pigs for lasting 28 days (74). Plant essential oil (PEO) improves the growth performance and intestinal mucosa growth in weaned pigs, contributing to mediate the improvement in intestinal integrity and function (75). *Capsicum oleoresin* (CAP) and *garlic botanical* (GAR) are natural products and widely used in foods and drugs. Feeding CAP and GAR increases the expression of genes related to the integrity of membranes in infected weaning pigs and enhances gut mucosa health, which results in ameliorating the diarrhea and clinical immune responses in infected pigs fed plant extracts (76). Supplementation of essential oil from Brazilian *red pepper or chlorohydroxyquinoline* in weanling pig diets affects gut microbiota and histology but not affecting performance and organ weight, and high doses of essential oil can reduce the incidence of diarrhea (77). Piglets fed the *Sangrovit* (SAG) diet have an average lower value for crypt depth of the jejunum and greater values for villus height in the ileum and ratios of villus height to crypt depth in the jejunum and in the ileum. SAG can potentially improve the intestinal morphology and modify the intestinal luminal environment (78). Rotavirus (RV) impairs intestinal morphology, antioxidant capacity, and microbiota, and increases apoptosis of jejunal epithelial cells of in piglets. Dietary *Lentinan* (LNT) supplementation is found to improve intestinal morphology, permeability, and apoptosis of jejunal epithelial cells in piglets (79). OEO can induce a higher glycoconjugate production in the gut, creating a physical barrier against microorganisms. *OAE* (an aqueous extract of oregano) supplementation improves the production of glycoconjugates, enhances the protection of intestinal mucosa in pig gut (80). Rosemary (*Rosmarinus officinalis L*.) extract (RE) has multiple pharmacological and biological activities, and has been used as a food additive. Piglet supplementation with RE shows longer villus height and villus height/crypt depth in the jejunum and ileum, in addition to a lesser crypt depth in the jejunum and ileum, which can improve growth performance, nutrient digestibility, and intestinal morphology in weaned piglets (81). Cactus (*O. ficus indica*) intake does not affect piglet development in lactating sows, but a higher length of intestinal villi in the jejunum and transverse portion to improve live weight in post-weaning piglets (82).

The OEO-treated pigs show decreased endotoxin levels in serum and increased villus height and the expression of Occludin and Zonula occludens-1 (ZO-1) in the jejunum (83). The main active compounds *carvacrol* and *thymol* in OEO contribute to have antimicrobial and antioxidative capacity to improve intestinal barrier function and growth in pigs (62). In addition, supplementation of AGP or microencapsulated *organic acids* (OA) and EO does not significantly attenuate the induced inflammation, reduce digestive enzyme activities, and elevate gut permeability in piglets infected with *Escherichia coli* F4 (ETEC F4) in weaned piglets (84). However, the herbal extract mixture (HEM), a mixture of golden-and-silver honeysuckle (*Lonicera japonica Thunb*), huangqi (*Astragalus menbranaceus*), duzhong leaves (*Eucommia folium*) and dangshen (*Codonopsis pilosula*), had no effects on growth performance and organ weight of weaned pigs. However, compared with the control group, HEM can improve intestinal morphology and elevate the expression of nutrient transporters in the ileum (85). Practices have focused on supplementing in weaning piglets with phytochemicals to alleviate weaning complications. Some benefits are observed with supplementation of phytochemicals, and further research explored the potential of maternal supplementation during fetal and postnatal life to support homeostasis in the piglet gut.

### Effect of Phytochemicals on Gut Microbiota

Intestinal health is related to transform of the intestinal microbiome composition, leakage of the mucosal barrier, and/or inflammation, which is determined by host, nutritional, microbial, and environmental factors (86). The dynamic gut microbiota of mammals contributes to regulating immune responses, engaging in the metabolism of dietary nutrients, and constituting a resistance line against pathogenic bacteria infection (87). Gut microbiota shifts or dysbiosis are accompanied with colitis and infectious diseases (88, 89).

Gut microbiota has a crucial role in intestinal absorption and digestion, intestinal immune regulation, and intestinal health of weaned piglets (38). Gut bacterial dysbiosis usually results in post-weaning diarrhea (18, 90, 91), which lead to intestinal barrier injury, reduce the size of the intestinal villi, result in atrophy of the enterocytes and lower digestive capacity, and decrease the weight gain of the piglet (82, 92). Intestinal tract is an important target of nutritional regulation. Strategies that modulate the microbiota and intestinal integrity of the pig in a favorable way should be sought. It is demonstrated that *Firmicutes* and *Bacteroidetes* are the most abundant phyla in the fecal microbiota of piglets, accounting for more than 90% of the fecal bacterial community at both pre-weaning and post-weaning periods (72). *Coix* seed supplement significantly increases the abundance of phylum *Bacteroidetes* and genus *Lactobacillus*, and reduces the abundance of phylum *Prevotella* in the gut microbiota, which could be associated with growth performance in weaned piglets (26). Dietary supplementation with *tannic* acid (TA) elevates the expression of nutrient transporter genes such as *solute carrier family 15, member 1 (SLC15A1)* and *solute carrier family 6, member 19 (SLC6A19)* of the ileum in weaning piglets. The relative abundances of *Firmicutes* at the phylum level are deceased. However, TA elevates the relative abundances of *Bacteroidetes* at the phylum level, *Bacteroidia* at the class level, and *Bacteroidales* at the order level (38). Herb of purple loosestrife (Lythrum salicaria L. from Lythraceae family) (LSH) has no significant influence on microbiota diversity and metabolic activity, but is able to modulate the gut microbiota composition, which could be applied as therapy or prevention of post-weaning diarrhea in piglets (93). *Cortex Phellodendri Extract* (CPE) also has a positive effect on diarrhea in weaning piglets. CPE can inhibit the growth of harmful intestinal bacteria including *Escherichia* and *Shigella*. Meanwhile, the composition of gut microbiota, the diversity, and evenness of gut microflora have been elevated (94). Dietary *oleum cinnamomi* (OCM) and *marine* macroalgal extracts supplementation modulate the intestinal microbiota and improve intestinal function in weaned piglets (72, 95). *Grape seed proanthocyanidins* (GSPs) with half-dose colistin are equivalent to antibiotic treatment and assist weaned animals in resisting intestinal oxidative stress by increasing diversity and improving the balance of gut microbes. Dietary GSPs result in increasing concentrations of propionic acid and butyric acid to activate GPR41, which may in turn contribute to improve the intestinal mucosa barrier, decrease intestinal permeability, improve intestinal morphology, reduce the occurrence of diarrhea (96, 97).

Besides the weaned piglets, the gut microbiota modulated by phytochemicals also contributes to pigs at other growth stages. Addition of 1.5% bamboo vinegar powder promotes the growth and development of growing-finishing pigs, increases the abundance of *Firmicute*/*Bacteroidetes*, enhances the ability of the host to absorb food energy and store more body fat in 37-days experiments. Additionally, bamboo vinegar powder has positive effects on promoting the abundance of *Lactobacillus* and *Thalassospira* and on inhibiting *Streptococcus* and *Prevotella* growth (98). 1% Wakame seaweed powder maybe alter intestinal microflora preferentially, namely, which are observed that an increase in *Lactobacillus* and a decrease of *Escherichia coli* to improve the gut health and immunity of pigs (99). Feeding the diet supplemented with dried *Jerusalem artichoke* (DJA) tubers, as a source of inulin-type fructans, affects the microbiota activity in the large intestine by decreasing proteolytic fermentation and detrimental bacterial enzyme activity. 4% of DJA tubers modifies the microbiota ecology in the large intestine of young pigs to a greater extent than 2% of inulin from chicory root and the applied probiotics do not enhance the effects of prebiotics (100). Additionally, supplementing with OEO during lactation increases the relative abundance of *Lactobacillaceae* family and *Fibrobacteriaceae* and *Akkermansiaceae* in sows. However, analysis of piglet microbiota reveals a relative decrease in *Enterobacteriaceae*, nevertheless butyrate producers (*Lachnospiraceae* family) are increased at two weeks and four weeks of age (62). Together, phytochemicals can increase the abundance of beneficial bacteria such as lactic acid bacteria, reduce the abundance of harmful bacteria such as *Escherichia coli* and *Streptococcus*, maintain the stability and balance of intestinal flora, regulate immune response, improve intestinal structure and nutrient metabolism, thus maintaining intestinal health and growth and development of pigs.

### Effect of Phytochemicals on Antiviral

It has been well documented that phytochemicals possess strong antiviral action against several DNA and RNA viruses (60, 101). The effective and efficient properties of pure natural products are a novel source of rich antiviral drugs, which exerts antiviral strategy in animal infectious disease.

Phytochemicals differently regulate the expression of genes related to immunity in alveolar macrophages of PRRSV (porcine reproductive and respiratory syndrome virus)-infected pigs (102). *Curcumin* can block PRRSV internalization and virus-mediated cell fusion *in vitro* (103), and *Xanthohumol* inhibits PRRSV proliferation and alleviates oxidative stress induced by PRRSV (104). *Sugar cane* extract (SCE)-treated pigs show a significant enhancement of natural killer cytotoxicity, lymphocyte proliferation, phagocytic function of monocytes, and interferon-gamma (IFN-γ) production of CD4^+^ and gammadelta T cells compared with the controls after pseudorabies virus (PRV) infection, which may be extensively applied in field for the prevention of infections (105). A study investigates that different concentrations of *Radix isatidis polysaccharide* can inhibit pseudorabies virus (PRV) replication by 14.674-30.840%, prevent infection at rates of 6.668-14.923%, and kill this virus at rates of 32.214-67.422% (106). Porcine epidemic diarrhea virus (PEDV) causes lethal diarrhea in suckling piglets. Even commercial vaccines do not guarantee effective protection against PEDV. It is urgent to find alternatives to antibiotics to prevent diarrhea in the context of anti-drug prohibition. Isoflavonoid is the major component of *Puerarin* (PR), which is isolated from the Chinese herb Gegen and possesses anti-inflammatory and antiviral activities. PR (0.5 mg/kg body weight between days 5 and 9) plays antiviral and anti-inflammatory roles in piglets infected with PEDV (10^4.5^ TCID_50_ per pig on day 9) *via* regulating the interferon and NF-κB signaling pathways both *in vitro* and *in vivo*. In addition, *Tomatidine* inhibits PEDV replication mainly by targeting 3CL protease (107, 108). However, formic acid has no function in any anti-PEDV activity in the early steps of the viral cycle with the only exception of a slight effect of viral inactivation at the lowest PEDV MOI. Formic acid at a dose of only 1,200 ppm can effectively inhibit PEDV replication in Vero cells (109). However, the combination of essential oil and benzoic acid enhances the degradation of PEDV RNA (110). Rotavirus (RV) impairs intestinal morphology, antioxidant capacity, and microbiota, and increases apoptosis of jejunal epithelial cells of in piglets. It is reported that *lentinan* (LNT) relieves RV-induced diarrhea in piglets, which can be due to the increase in antioxidant capacity, reduction in apoptosis and improvement of the microbiota-increased gut barrier (79). Porcine circovirus type 2 (PCV2) often causes multiple system failure in nursery pigs. *Cepharanthine and Curcumin* can inhibit mitochondrial apoptosis induced by PCV2 by through increasing Bcl-2, reducing Bax, caspase-3, ROS, and decreasing mitochondrial membrane potential (MMP) (111). *Selenizing astragalus polysaccharides* (sAPS) attenuates oxidative stress-induced PCV2 replication promotion through inhibition of autophagy *via* the increased phosphorylation of Akt and mTOR (112). Additionally, PCV2 induced immunosuppression and total flavonoids of *Spatholobus suberectus Dunn* (TFSD) might be able to protect animals from virus infection *via* regulation of immune function and inhibition of oxidative stress (113). Additionally, an essential oil blend of three oils, *E. globulus*, *P. sylvestris*, and *L. latifolia*, can enhance the pig humoral immune system by enhancing IgG levels and reducing IgM levels, suggesting that this novel formulation functions as a potential agent to minimize African swine fever virus (ASFV) transmission in an *in vivo* trial in swine (114). To sum up, inhibition of virus replication in different ways may be the main advantage of phytochemicals and can be supplemented by alleviating oxidative stress, regulating interferon expression, reducing cell apoptosis and improving the intestinal barrier. Therefore, these results demonstrate that phytochemicals exert promise as a new antiviral drug in the future.

### Effect of Phytochemicals on Antimicrobial Activity

Phytochemicals possess strong potential as antimicrobial agents. The antimicrobial action of essential oils not only depends on phytochemical constituents but also interactions among different components leading to synergistic or antagonistic activities (60). The plant extracts containing nonpolar compounds such as *α-amyrin*, *friedelan-3-one*, *lupeol*, and *β-sitosterol*, damage the internal and external anatomy of the cytoplasmic membrane and inner structure, and lead to an increased influx of propidium iodide into treated bacterial cells to exert antimicrobial and anti-inflammatory properties (28).

*Thymol* activity is related to alterations in the bacteria cell wall, but cinnamaldehyde activity is associated with the cell wall of both enterobacteria and protein and fatty acid changes for *C. perfringens* (115). In addition, an additive effect is shown for *carvacrol-oregano* essential oil for *Escherichia coli*, formic acid-*carvacrol* and formic acid-*thymol* for *Salmonella* spp. and *carvacrol-cinamaldehyde* for *C. perfringens* (29). *Ginseng polysaccharides* (GPS) (200 mg/kg) supplementation improves immunity-related biomolecular levels in sow serum and milk during late pregnancy and lactation, which may be further beneficial to piglet health and growth through biological transmission effects (116). In a lasting 28-days experiment, water extract of *Artemisia ordosica* (WEAO) supplementation improves the apparent nutrient digestibility of piglets in a linear or quadratic dose‐dependent manner. In addition, dietary WEAO quadratically increases serum concentrations of IL‐1, IL‐4, TNF‐α, soluble surface antigen CD8 (sCD8), immunoglobulins (Ig)‐A and linearly increased serum concentrations of IL‐2, IL‐6, IgG, IgM. Furthermore, dietary WEAO linearly or quadratically decreases serum concentrations of malondialdehyde but quadratically increases the activities of antioxidant enzymes and total antioxidative capacity (117).

Enterotoxigenic *Escherichia coli* is considered one of the main causes of diarrhea in weaning piglets. 2% (20 g/kg) *chestnut* extract (CE) can represent a promising alternative to antibiotics immediately after weaning for improving growth performance and reducing post-weaning diarrhea (PWD) caused by *ETEC F4* (118). *Anethole* (300 mg/kg) coated with corn starch can improve the growth performance of weaned piglets infected by *Escherichia coli* K88 through decreasing the expression of TLR5, TLR9, MyD88, IL-1β, TNF-α, IL-6, and IL-10 in the jejunum, attenuating intestinal barrier disruption and enriching the abundance of beneficial flora in the intestines of the piglets (119). Acetone crude leaf extracts of *Syzygium* and *Eugenia* (*Myrtaceae*) have good antimicrobial activity as well as a protective role on intestinal epithelial cells against enterotoxigenic *E.coli* bacterial adhesion (120). Plant-derived natural steroid compound *phytosterol* (PS) supplementation exerts similar effects on growth, anti-inflammation and intestinal microorganisms as supplementation with polymyxin E in piglets. Despite of no significant effect on growth, 0.2 g/kg PS supplementation activates CD4^+^ T cells and results in a Th2 shift, which suggests that PS can improve immunity and anti-inflammatory activity and ameliorate diarrhea in weaned piglets (121). *Baicalin–aluminum* complex (BBA) administration alters the structure of the gut microbiomes. Then the diarrhea rate reduces significantly after treatment with BBA in piglets (122). *Scutellaria baicalensis* extracts (SBE) supplementation can potently attenuate diarrhea in weaning piglets and decrease inflammatory cytokine expression through inhibiting the NF-κB and P38 signaling pathways, attenuating *Escherichia coli* K88-induced acute intestinal injury in weaned piglets (123).

*H. parasuis* infection provokes the expression of cytokines and pathways activation, and induces the release of high-mobility group box 1 (HMGB1). HMGB1 protein is related to the pathogenesis of various infectious pathogens. Baicalin displays important anti-inflammatory and anti-microbial activities. Baicalin significantly reduces the release of HMGB1 in peripheral blood monocytes induced by *H. parasuis* (124). Tea polyphenols can inhibit *H. parasuis* growth in a dose-dependent manner and attenuate the biofilm formation of *H. parasuis*. In addition, tea polyphenols inhibit the expression of *H. parasuis* virulence-related factors. Moreover, tea polyphenols can confer protection against a lethal dose of *H. parasuis* and reduce pathological tissue damage induced by *H. parasuis* (125). Together, some plants are known as nutraceuticals because they exhibit antimicrobial properties for susceptible in pig production. These activities of phytochemicals may be achieved by rebuilding the structure of bacterial cell membranes and cell walls, inhibiting the expression of bacterial virulence factors, regulating the expression levels of inflammatory factors, and improving antioxidant enzyme activity.

### Effect of Phytochemicals on Immunomodulatory

Phytochemicals are hopeful alternatives to chemotherapeutics in animal production owing to their immunostimulant. Phytochemicals may activate the immunity of the host either by directly stimulating the innate immune system or through commensal microorganisms sustenance and pathogens inhibition (126, 127). The dietary administration of phytochemicals can modulate the following biological processes related to innate immune effector cells, namely “leukocyte activation”, “leukocyte activation involved in immune response”, “neutrophil activation”, and “neutrophil degranulation” (127). Neutrophils are rapidly recruited to infected tissues and can engulf bacteria directly or produce toxic antimicrobial mediators, which have always been considered as uncomplicated front-line troopers of the innate immune system equipped with limited proinflammatory duties (128).

Plants containing immunostimulatory properties improve the activity of lymphocytes, macrophages, and NK cells and thereby increase phagocytosis and stimulate interferon synthesis (129). Medicinal plants propose an alternate to conventional therapeutic strategies for numerous ailments, particularly when suppression of inflammation is expected (130, 131). It has been reported that dietary supplementation of phytochemicals increases the number of white blood cells and other immune parameters and possesses significant anti-inflammatory activity (132). Chinese medicinal herbs (CMH) supplementation composed of *Panax ginseng*, *Dioscoreaceae opposite*, *Atractylodes macrocephala*, *Glycyrrhiza uralensis*, *Ziziphus jujube* and *Platycodon grandiflorum* can enhance the immune activities of polymorphonuclear leucocytes (PMNs), and reduce diarrhea frequency in weanling pigs. (133). ORE treatment alters the lymphocyte proportion and the ratio of CD4^+^ and CD8^+^ T-cells in the serum of non-stimulated and in LPS-stimulated piglets (134). NF-κB is downregulated on peripheral blood mononuclear cells (PBMCs) in outdoor reared pigs when fed the ORE diet (135). Leukocyte populations in blood reveal that the percentages of Th lymphocytes, γδ T lymphocytes, and B lymphocytes are more affected by body weight in weanling piglets. The percentage of this leukocyte population is higher in high birth weight (HBW) piglets receiving CKTL + COL diet [a cocktail of feed additives containing cranberry extract, encapsulated carvacrol, yeast-derived products, and extra vitamins A, D, E, and B complex (CKTL); CKTL diet with bovine colostrum in replacement of plasma proteins (CKTL + COL)] when compared with low birth weight (LBW) piglets of the same group (136). The *E. coli* challenge tends to increase the number of total WBC on d 5 and increase it on d 11 post-infection compared with the sham group, but the plant extracts (10 ppm of *capsicum oleoresin*, 10 ppm of *garlic botanical*, or 10 ppm of *turmeric oleoresin*) treatments in the *E. coli*–challenged group decrease total WBC and the number of neutrophils (137), and feeding each of the plant extracts has effects on expression of several genes that are counter to the effects of *E. coli* (76). Additionally, *Agrimonia procera* (AP) increases the immune response in LPS-treated piglets (138). SBE supplementation can potently attenuate diarrhea in weaning piglets and decrease inflammatory cytokine expression through inhibiting the NF-κB and p38 pathways (123). Moreover, dietary supplementation with isoquinoline alkaloids extracted from *Macleaya cordata* R. Br. boosts the immune system, regulates the metabolic process and finally promotes growth and development in swine (139). The immunomodulatory effects of plant-derived compounds are increasingly attracting people’s interest. Studies that applied plant extracts with known components, or single constituents, and de facto measured parameters with the relevant to immune function as outcome measures are included (140). Several studies proving to have effects on weaned piglet immune regulation apply a ready-to-use plant product. The immunomodulatory effects are mainly inspected on a cellular (monocytes/macrophages, neutrophil granulocytes, NK cells, and T and B lymphocytes) and molecular level (cytokines, immunoglobulins), data on the potential molecular genetical mechanisms accounting such immunological effects in the literature is limited (140).

### Effect of Phytochemicals on Detoxification of Mycotoxins

Deoxynivalenol (DON), zearalenone (ZEN), and aflatoxin B1 (AFB1) are the main mycotoxins that frequently contaminate maize and grain cereals which are the main feedstuff in pig diets (141). The effects of toxic include immune modulation, disruption of intestinal barrier function, and cytotoxicity leading to cell death, which all result in impaired pig performance and impose risks to the health of both humans and animals (142, 143). It is reported that the genera *Lactobacillus* (particularly in DON) and *Bacteroides* dominate the bacterial flora in both the DON and ZEN dietary treatments, and there may be potential opportunities to isolate and characterize useful probiotics that decrease the level of mycotoxins (143). However, phytochemicals may potentially reduce tissue damage mediated by mycotoxins by regulating immune function, improving the abundance of intestinal flora, and decreasing oxidative stress.

Liver is the earliest target for AFB1 toxicity in both humans and animals (144). Grape pomace is able to reduce the gastrointestinal absorption of mycotoxins (145). Dietary *grape seed meal* (GSM) can mitigate the AFB1-induced toxicity in pigs (146). Diet containing 8% GSM (grape seed meal) by-products ameliorates histological liver injury and oxidative stress in the liver of the pig after weaning exposed to AFB1 through the contaminated feed for 28 days, accompanied by decreasing MAPK (mitogen-activated protein kinase) signaling pathway and inhibition of NF-κB signaling pathway by AFB1 diet (144). After 30 days of experiment, GSM (8% inclusion in the diet) and AFB1 (320 g/kg feed) increase the relative abundance of phylum *Bacteroidetes* and *Proteobacteria*. However, compared to the individual treatments, the *Firmicutes* abundance is decreased in a synergic manner. An additive or synergistic action of the two treatments were identified for *Lactobacillus*, *Prevotella*, and *Campylobacter*. On the contrary, a rather antagonistic effect is observed on *Lachnospira* (147). Additionally, *Dihydromyricetin* (DHM) alleviates cell injury induced by DON and it is possibly through its antioxidant activity, anti-inflammatory activity, or ability to regulate metabolic pathways (148). Astragalus polysaccharides (APS) can attenuate the immune stress induced by *ochratoxin A* (OTA) *in vitro* and *in vivo via* activation of AMPK/SIRT-1 signaling pathway (149). Plant derivatives can be used to diminish the detrimental effects caused by mycotoxins in pigs, more specifically in the gastrointestinal tract (141). *Phytobiotics*, such as plant extracts, typically include bioactive extracts and characterize with antioxidative and anti-inflammatory properties (150). *Phytobiotics* are often included in mycotoxin-detoxifying agent formulas to mitigate the negative effects of multiple mycotoxins in diets fed to pigs, which is directly related to the gut health and gut microflora of pigs (151). In summary, the effects of phytochemicals on mycotoxin poisoning can be achieved by regulating signaling pathways, improving histological damage, oxidative stress, flora abundance, and regulating anti-inflammatory activity and metabolic activity.

## The Action Mechanisms of Phytochemicals

Phytochemicals exhibit antimicrobial activity, antioxidative capacity, anti-inflammatory activity, and other properties against pathogenic microorganisms (bacteria, fungi, and viruses) or risks. Although the chemical composition and function attributes of phytochemicals have been studied previously, the comparison of their chemical constituents using different extraction methods as well as the linked mechanisms still remain unexplored (152). A number of mechanisms have been proposed and proven. The studies have focused on enzymatic activity, endocrine disruption, plasma membrane homeostasis, singling pathway. In this manuscript, the antimicrobial, antivirus, antioxidant, and immune regulation mechanisms of phytochemicals are mainly discussed.

### Antimicrobial and Antivirus Mechanism of Phytochemicals in Pig Production

It is demonstrated that numerous antimicrobial phytochemicals have been discovered or better characterized (22, 153). Wang et al. summarizs the antimicrobial mechanisms of ginseng, one of a phytochemicals (a) inhibit the microbial motility and quorum-sensing ability; (b) affect the formation of biofilms and destroy the mature biofilms, which can weaken the infection ability of the microbes; (c) perturb membrane lipid bilayers, thus causing the formation of pores, leakages of cell constituents and eventually cell death; (d) active of the host immune system and attenuate microbes induced apoptosis, inflammation, and DNA damages, which can protect or help the host fight against microbial infections; and (e) inhibit the efflux of antibiotics that can descend the drug resistance of the microbial (154). Besides *ginseng*, *cinnamon* extracts have been reported to inhibit bacteria by damaging cell membranes, altering the lipid profile, inhibiting ATPases, cell division, and biofilm formation (155). *Carvacrol* can cross the bacterial plasma membrane and bind molecules such as ATP or monovalent cations such as *K* ^+^ and transport them out of the bacterial cell depending on a phenolic OH group, seriously altering the membrane potential and homeostasis (22, 156). *Thymol* has the ability to incorporate into the polar-head group region of the lipid bilayer, altering the structural integrity and fluidity of the membrane through hydrogen bonding and hydrophobic interactions exerting antimicrobial activity (157, 158). *Cortex Phellodendri Extract* (CPE) has a positive effect on diarrhea in weaning piglets. The mechanism behind these effects is that CPE can diminish the adhesion of ETEC to intestinal epithelial cells by suppressing the expression of the bacterial flagellum genes (94). Furthermore, *Cranberry* extract (20 μg or 100 μg/mL) inhibits *in vitro* adhesion of F4^+^ and F18^+^ *E. coli* to pig intestinal epithelium and reduces *in vivo* excretion of pigs orally challenged with F18^+^ verotoxigenic *E. coli* (159).

Many plant-derived natural substances are potential keys to designing antiviral therapies for inhibiting viral replication and infection (160, 161). The main active ingredients of Chinese medicines or phytochemicals such as *quercetin, kaempferol, luteolin, isorhamnetin, baicalein, naringenin*, and *wogonin* could target on AEC2 and 3CL protein of COVID-19. They exert the antivirus, inhibiting inflammatory mediators, regulating immunity, and eliminating free radical properties through the signaling pathways of IL-17, arachidonic acid, HIF-1, NF-κB, Ras, and TNF (162). Pterodontic acid is isolated from *L. pterodonta* and shows selective antiviral activity of influenza A virus, which is most probably associated with inhibiting the replication of influenza A virus by blocking nuclear export of viral RNP complexes, and attenuating the inflammatory response by inhibiting activation of the NF-κB pathway (163). Isoflavonoid is the major component of *Puerarin* (PR), which is isolated from the Chinese herb Gegen and possesses anti-inflammatory and antiviral activities. PR supplementation inhibits PEDV-induced NF-κB activation, demonstrating that PR has the potential to be an effective antiviral feed additive (108). PRRSV is one of the major swine pathogens. Tea seed saponins (TS) inhibit PRRSV-induced cell apoptosis and effectively suppress PRRSV replication by reducing the expression of the host cellular gene PABP, and significantly restrain virus N gene/protein expression (164). *Xanthohumol*, a prenylated flavonoid found in hops, significantly suppresses PRRSV proliferation by activating the Nrf2-HMOX1 pathway, which may be helpful for developing a novel prophylactic and therapeutic strategy against PRRSV infection (104). Studies showed that APS attenuated PCV2 infection by inhibiting oxidative stress and endoplasmic reticulum stress and by blocking NF-κB pathway (165, 166), and *selenizing astragalus polysaccharide* (sAPS) attenuates PCV2 replication promotion through autophagy inhibition *via* PI3K/AKT activation relying on Akt and mTOR phosphorylation (112). *Aloe* (Ae) extract can hamper completely the proliferation of PEDV in Vero and IPEC-J2 cells *in vitro*, and can reduce virus load and pathological changes in the intestinal tract of pig challenge with highly pathogenic PEDV variant GDS01 infection. However, Ae could not degrade S1 protein *in vitro*, indicating that Ae may directly inactivate the virus infectivity, but no effect on virus nucleic and virus invasion (167). Virus-infected cells release signals to recruit and activate immune cells. These immune cells secrete a variety of cytokines and chemokines to recruit more immune cells to the lesion site (168). Phytochemicals can be beneficial for animal health and considered as an alternative agent for antimicrobial and antivirus therapy. Although, potential targets and pathways of phytochemicals prescriptions were predicted through network pharmacological analysis. Antimicrobial and antivirus mechanisms of phytochemicals in pig production need further to be investigated.

### Antioxidant and Immune Regulation Mechanisms of Phytochemicals in Pig Production

The main antioxidant and anti-inflammatory mechanisms found in phytochemicals are largely attributed to the regulation of signaling pathways. The Nrf2-ARE signaling is an important mediator of cellular response during an oxidative stress condition. Nrf2 may be considered as a key factor that protects cells against oxidative damage and contributes to the survival of the cell (169). *Tagetes erecta* flowers essential oils exerting antioxidative stress, anti-apoptotic and anti-inflammatory effects may rely on Nrf2/HO-1 and NF-κB signaling pathways (170). *Isoegomaketone* (IK), an essential oil component of *Perilla frutescens* (L.) *Britt*, inhibits NO production through simultaneous induction of HO-1 and inhibition of the IFNβ-STAT-1 pathway possessing anti-inflammatory activity (171).

NF-κB is a critical regulator of immunity and responsible for the transcription of the gene encoding many pro-inflammatory cytokines and chemokines (172). NF-κB activation results in tissue changes characteristic of inflammation. *Schisandra chinensis* extract (SCE) and *Schisandra chinensis* lignans (SCL) can greatly inhibit the TLR4/NF-κB/IKKα signaling pathway, and indicate that *Schisandra chinensis* ameliorates depressive-like behaviors by regulating microbiota-gut-brain axis *via* its anti-inflammation activity (173). *Astilbe Chinensis ethanol* extracts (ACE) inhibit LPS or thioglycollate (TG)-induced inflammation by blocking the NF-κB signaling pathway in macrophages (174). Additionally, MAPK and protein kinase A (PKA) signaling pathways are involved in antioxidant and immune regulation (175).

**Figure 1:**
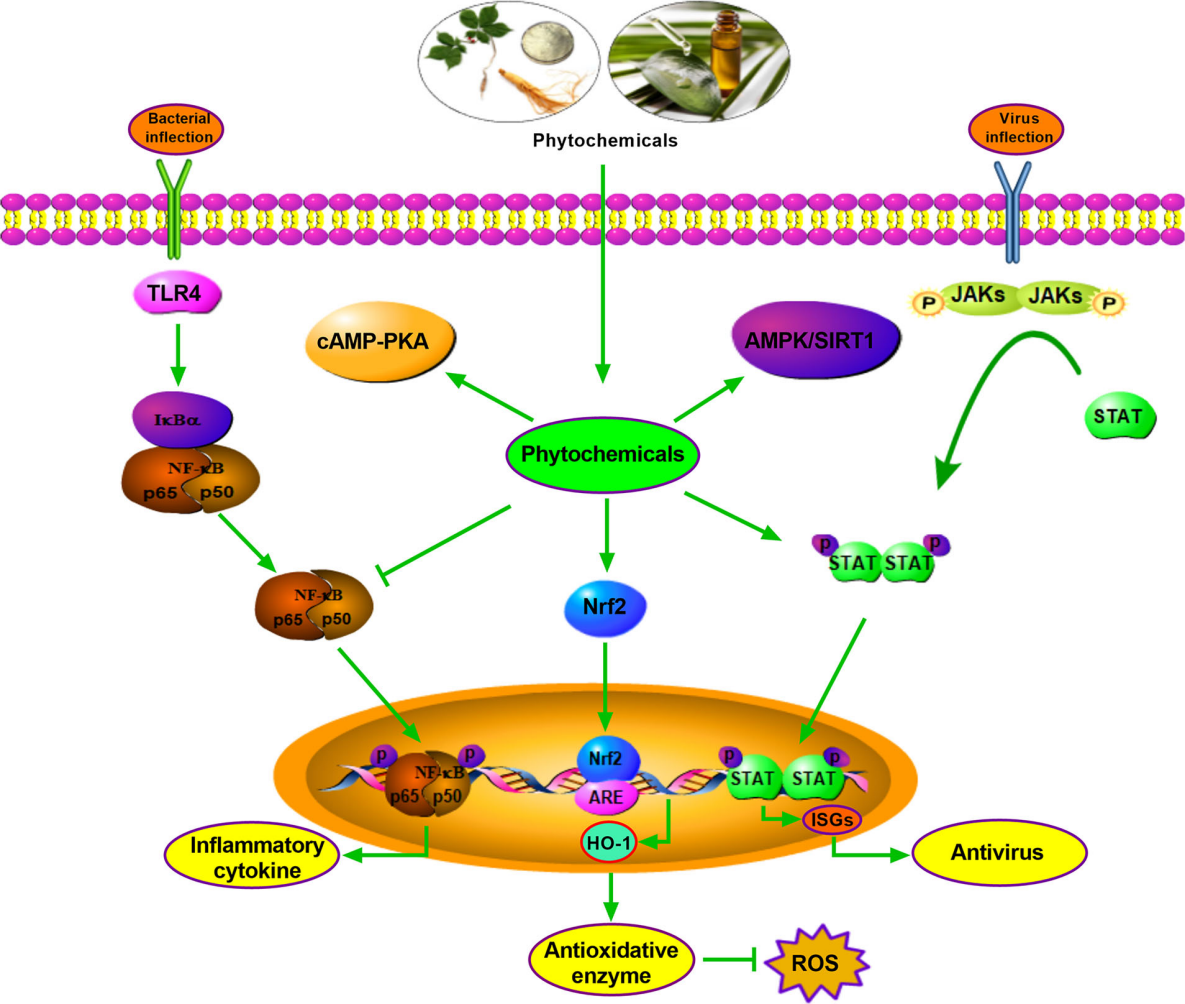
Antimicrobial, antivirus, antioxidant mechanism of parts phytochemicals. Phytochemicals exert antimicrobial effect by inhibiting NF-kB signaling, and function as antioxidant role by activating Nrf2-HO-1 pathway. Phytochemicals show antivirus function relying on STAT pathway. Other pathways such as cAMP-PKA and AMPK/SIRT1 are also involved in antimicrobial, antivirus or antioxidant effects.

OEO is generally attributed to its antimicrobial and anti-inflammatory effects and contributes to promoting intestinal barrier integrity in pigs. The protective effect of OEO on the intestine is associated with the decrease of intestinal *E. coli* population and the inactivation of the JNK, ERK1/2, Akt, and NF-*κ*B signaling pathways (83). *Illicium verum* extracts (IVE) and probiotics with added glucose oxidase (PGO) promote antioxidant capacity through upregulating the hepatic and jejunal Nrf2/Keap1 pathway of weaned piglets. Therefore, IVE and PGO can be recommended as a new potential alternative to antibiotics in piglets’ diets (176). *Ulva prolifera* extracts contain multiple functional active substances, which relieves oxidative stress via Nrf2 signaling, but not through AMPK pathway in weaned piglets treated with hydrogen peroxide (177). It is shown that SBE supplementation can potently attenuate diarrhea in weaning piglets and decrease inflammatory cytokine expression through inhibiting the NF-κB and P38 signaling pathways (123). Additionally, KEGG Pathway enrichment analysis by RNA sequencing shows that PI3K-Akt, AMPK, Rap1, and peroxisome signaling pathways are enriched in Tibetan pig liver after Chinese wolfberry (*Lycium barbarum*) and Astragalus (*Astragalus membranaceus*) extract treatment, and the plant extracts exert antioxidant and anti-stress ability (178). *In vitro*, OEO induces SOD1 and GSH expression through Nrf2 activation and alleviates hydrogen peroxide-induced oxidative damage in IPEC-J2 Cells (179). A low dose of *eugenol* (100 μM) improves selectively permeable barrier function during LPS-induced inflammation and tends to increase TEER values in the IPEC-J2 cell line (180). The antioxidant and immune regulation effects of phytochemicals are partly related to the activation of signaling pathways. These facts justify the urgency for therapeutic alternatives in pig production, preferentially combining selective antioxidant and anti-inflammatory activities, which may be efficiently to promote growth performance. Although numerous antioxidant and anti-inflammatory phytochemicals have been discovered or better characterized, in most cases, their mechanisms of action still have been clarified (**Figure 1**).

## Discussion and Prospect

Scientific feeding strategies need to be considered to achieve certain goals in pig production. It is imperative to develop by-products or feed additives with high nutritional value, low cost, and easy access, to solve the shortage of feed resources, alleviate the current situation of human and animal competition for food, reduce drug resistance and environmental pollution, and provide safe meat. In the application of feed additives to improve animal performance and health, the chemical composition of feed additives, the mechanism of action, the release efficiency of the target of action, the relationship between feed and additives, nontoxic side and no residue, are important factors that should be considered. Plants are the source of a large proportion of medicines. Phytochemicals are considered to have medicinal properties possessing a wide variety of bioactivities including antimicrobial, antiviral, antioxidant, anti-inflammatory (181). Some novel biological functions including medical and commercial benefits of some phytochemicals have been fully explored, which can be acted as an alternative to antibiotics (182, 183).

Antibiotics have played a crucial role in the growth and development of the swine industry. Their efficiency in increasing growth rate, improving feed utilization, and reducing mortality from clinical disease is well documented (184). However, antibiotic residues, drug resistance, and environmental pollution seriously threaten human health and the sustainable development of the pig industry. It is necessary to improve nutritional programs in swine diets using phytochemicals, which contributes to increasing production efficiency and profitability, and reducing the environmental impact of pork production. The prohibition of antibiotics in pig feed provides a good application prospect for the development of effective phytochemical additives (**Table 1**). The principles of choosing by-products or additives are that they do not require more time or effort to process (188). However, there is limited information available on the pharmacodynamics/kinetics of phytochemicals in pig production. Firstly, efficient extraction methods to obtain active ingredients may be a pending challenge for many phytochemicals. It is imperative to use modern technologies including supercritical fluid extraction, subcritical extraction, liquid or solvent-free microwave extraction to obtain the effective substances. Secondly, the morphological damage, toxicity, and effectiveness, as well as the dosage in animals need to develop new techniques and to evaluate. Furthermore, the potential pharmacological interactions of ingredients and action mechanisms need to be further investigated. Importantly, the quick growth rate, stress-load effects of combinational feed additives, and cost/benefit analysis can be conducted to understand the achievable application of phytochemicals in pig production. Besides, careful consideration of group size, sample size, and how these factors may influence study outcomes, protocol implementation, sample collection, and recording of important information will be beneficial to the research progress of effective alternatives of antibiotics (189). Some studies of phytochemicals in pig production indicate the fruitfulness but others are still unclear. Experiments are needed to evaluate its economic aspects and efficacy under clinical conditions, so that phytochemicals can be used as natural medicine alternatives to antibiotics in the pig industry.

**Table 1:** Summary of the application of part phytochemicals in pig production.

Compound	Animal category/cell	Dosage	Supplemented ways	Major biological functions	Experimental duration	Reference
Acetone crude leaf extracts of Syzygium and Eugenia (Myrtaceae)	Intestinal epithelial cells	10 mg/mL	Added to cells directly	Antibacterial, protecting intestinal epithelial cells against enterotoxigenic *E. coli* bacterial adhesion	60 min	(120)
Anethole	Weaning piglets	300 mg/kg	Coating with corn starch	Anti-inflammatory, attenuating intestinal barrier disruption, enriching the abundance of beneficial flora, improving growth performance	19 d	(119)
Aqueous neem fruit extracts	Grower pigs	25%	Spraying extracts with organic liquid soap	Antiparasitic	Once a week for 6 weeks	(185)
Astragalus polysaccharide	Boar sperm	0.25, 0.5, 0.75, 1 mg/mL	Added to the basal medium	Preserving sperm motility, acrosome integrity and mitochondrial membrane potential, improving antioxidant capacities, enhancing ATP levels	4°C, 10 d	(186)
Astragalus polysaccharide	PAM cell line, 3D4/21 cells	*in vitro* 20 μg/mL, *in vivo* 200 mg/kg	Administered APS in 0.5% CMC-Na by gavage	Attenuating the immune stress	60 h, 20 d	(149)
Baicalin–aluminum complexes	Piglets	5 mL contained BBA 1.36 grams	Huangqinsulv capsules	Altering the overall structure of the gut microbiomes, reducing the diarrhea rate	Intragastric administration for 3 d, twice a day	(122)
Baicalin	Piglet peripheral blood monocytes, Haemophilus parasuis *cell*	12.5, 25, 50, 100 μg/mL	Dissolved in and diluted with RPMI-1640 medium to treat cells	Reducing the release of HMGB1 in peripheral blood monocytes induced by *H. parasuis*	24, 36, 48 h	(124)
Bamboo vinegar powder	Growing-finishing pigs	1.50%	Diets are mixed with bamboo vinegar powder	Promoting the growth and development, enhancing the ability of the host to absorb food energy and store more body fat, improving bacteria abundance	37 d	(98)
Betaine	Finishing pigs	1250 mg/kg, 2500 mg/kg	Diets are supplemented with betaine directly	Promoting muscle fatty acid uptake, up-regulating genes related to fatty acid oxidation	42 d	(51)
Black pepper extract	Finishing pigs	0.025%, 0.05%, 0.1%, 0.2%, 0.4%	Diets are supplemented with extracts directly	Enhancing the growth performance, nutrient digestibility, fecal microbial, fecal gas emission, and meat quality	10 weeks	(50)
Cactus (*O. ficus-indica*)	Lactating sows	1%	Diets are supplemented directly	Increasing LIV in jejunum of weaning piglets, improving live weight	21 d	(82)
Chicory root inulin extract Dried chicory root inulin extract	Growing pigs	2% 4%	Diets are supplemented directly	Modulating energetic metabolism, increasing anti-oxidative capacity	40 d	(44)
Cultured wild ginseng root extracts	Boar sperm	2.0 mg/mL	Added to sperm directly	Improving male reproductive function, suppressing ROS production	1 h	(67)
Curcumin	Marc-145 cells and porcine alveolar macrophages	5, 10, 15 mM	Dissolved in DMSO to treat cells	Blocking PRRSV internalization and PRRSV-mediated cell fusion	1 h	(103)
Dihydromyricetin	IPEC-J2 cells	40 μM	Dissolved in PBS to treat cells	Antioxidant, anti-inflammatory, regulate metabolic pathways, alleviating cell injury induced by DON	24 h	(148)
Dried Jerusalem artichoke	Young pigs	4%	Diets are prepared as pellets of 4 mm diameter	Modifying the microbiota ecology in the large intestine	40 d	(100)
Forsythia suspensa extract Chito- oligosaccharide	Younger pigs	100 mg/kg 160 mg/kg	Diets are supplemented with extracts directly	Modulating intestinal permeability, antioxidant status and immune function, increasing performance	28 d	(74)
Garcinol	Sows in late gestation and lactation	200 or 600 mg/kg	Diets are supplemented with extracts directly	Improving the maternal health, antioxidative status, enhancing growth performance	From the 90^th^ day of pregnancy to day 21 postpartum	(47)
Ginseng polysaccharides	Late pregnancy and lactation	200 mg/kg	Diets are supplemented with extracts directly	Improving immunity	Late pregnancy and lactation	(116)
Grape seed meal	Weaning piglets	8%	Diets are supplemented with extracts directly	Ameliorating histological liver injuries and oxidative stress exposed to AFB1, improving bacteria abundance	28 d 30 d	(144) (147)
Lentinan	Weaning piglets	84 mg/kg	Added by replacing the same amount of corn starch in basal diet	Antioxidant, reducing apoptosis, improving gut barrier, relieving RV-induced diarrhea	19 d	(79)
Linseed oil	Sows in late gestation and lactation	3.50%	Diets are supplemented with extracts directly	Increasing immunoglobulins, modifying the fatty acid composition	Gestation of 107^th^ d to the lactation of 28^th^ d	(52)
Murtilla extract	Boar sperm	0.0001 to 100 μg/mL	Added to sperm directly	Preserving sperm motility, acrosome integrity and mitochondrial membrane potential, improving antioxidant capacities, enhancing ATP levels	17°C, 30 min to 6 h	(8)
Oleum cinnamomi	Weaning piglets	50 mg/kg	OCM is well mixed with the basal diet	Modulating intestinal microbiota, improving intestinal function	20 d	(95)
Oregano essential oil	Pig(41.87 ± 1.23 kg	0.2%	Diets are supplemented with extracts directly	Help pigs tolerate the stress related to harsh, outdoor, rearing conditions	T1, 120 d T2, 190 d	(135)
Phytosterols	Weaning piglets	0.2 g/kg	Diets are supplemented with extracts directly	Improving immunity and anti-inflammatory activity, ameliorating diarrhea	27 d	(121)
Puerarin	Piglets	0.5 mg/kg BW	Dissolved in the liquid milk replacer	Exerting antiviral and anti-inflammatory effects infected with PEDV	5 d and 9 d	(108)
Radix isatidis polysaccharide	Swine testicle cells	0.625-0.078125 mg/mL	Added to cells directly	Inhibiting PRV replication, preventing infection, killing PRV	4 h, 24 h, 68 h	(106)
Resveratrol	IPEC- J2 cells	0, 25, 50 μM	Added to cells directly	Attenuating intestinal barrier injury	6 h	(65)
Scutellaria baicalensis extracts	Weaning piglets	1000 mg/kg	Prepared with maize starch and mixed in diet	Attenuating diarrhea, decreasing inflammatory cytokine expressions, attenuating *E.coli* K88-induced acute intestinal injury	14 d	(123)
Selenizing astragalus polysaccharide	PK-15 cells	20, 40 and 60 μg/mL	Prepared by the HNO_3_-Na_2_SeO_3_ method, then is added to plates directly	Attenuating oxidative stress-induced PCV2 replication	60 h	(112)
Tea polyphenols	Haemophilus parasuis cell	80, 160 and 320 μg/mL	Tea polyphenols is diluted with culture medium.	Inhibiting the biofilm formation and the expression of *H. parasuis* virulence-related factors, reducing pathological tissue damage	5 h, 16 h	(125)
Tea tree oil	Weaning pigs	100 mg/kg	Prepared with TTO, coating material and carrier, then added to diet	Benefits on growth performance, nutrient digestibility, antioxidative capacity and microbial community profile	0-28 d	(187)
Thymol	Piglets	25.5, 51, 153, 510 mg/kg	Microencapsulated in a lipid matrix	Regulating the integrity of the intestinal mucosa, anti-inflammatory, antioxidant	14 d	(64)
Tomatidine	Vero, ST, Marc-145, BHK-21, IPEC-J2 cells	2.5, 5, 10 μM	Dissolved in DMSO and added to plates	Inhibiting PEDV replication by targeting 3CL protease	30 min to 16 h	(107)
Wakame seaweed powder	From 20-30 kg to 70 kg	1%	Diets are supplemented with powder	Improving bacteria abundance, improving the gut health and immunity	24 d, 28 d, 48 d	(99)
Xanthohumol	Marc-145 cells and porcine alveolar macrophages	1 μM to 20 μM	Added to cells directly	Inhibiting PRRSV proliferation, alleviating oxidative stress induced by PRRSV	48 h	(104)

## Author Contributions

The idea for this article came from LL and HD. All authors contributed to structuring, drafting, writing, critically revising, and agreed to the published version of the manuscript.

## Funding

This work was sponsored by the National Natural Science Foundation of China(Grant: 32172808).

## Conflict of Interest

The authors declare that the research was conducted in the absence of any commercial or financial relationships that could be construed as a potential conflict of interest.

## Publisher’s Note

All claims expressed in this article are solely those of the authors and do not necessarily represent those of their affiliated organizations, or those of the publisher, the editors and the reviewers. Any product that may be evaluated in this article, or claim that may be made by its manufacturer, is not guaranteed or endorsed by the publisher.
